# Life’s Simple 7 and its impact on chronic bowel disorders: a study on constipation and diarrhea in the U.S. adult population

**DOI:** 10.3389/fmed.2025.1516210

**Published:** 2025-02-12

**Authors:** Hongzhi Sun, Lei Qi, Yiwei Ming, Weichen Wang, Maoneng Hu

**Affiliations:** ^1^The Third People's Hospital of Hefei, Hefei, Anhui, China; ^2^Hefei Third Clinical College of Anhui Medical University, Hefei, Anhui, China; ^3^The Second People's Hospital of Anhui Province, Hefei, Anhui, China

**Keywords:** Life’s Simple 7, chronic constipation, chronic diarrhea, bowel health, public health, lifestyle factors, NHANES

## Abstract

**Background:**

Chronic gastrointestinal disorders, such as chronic constipation and diarrhea, pose significant public health challenges, affecting quality of life and healthcare costs. Life’s Simple 7 (LS7), established by the American Heart Association, encompasses essential health behaviors that may influence bowel health.

**Methods:**

We utilized data from the National Health and Nutrition Examination Survey (NHANES) conducted between 2005 and 2010, focusing on adults aged 20 years and older. A total of 12,912 participants were included in the analysis. Bowel health was assessed through self-reported questionnaires, while LS7 was evaluated based on seven components: smoking status, physical activity, dietary quality, BMI, blood pressure, blood glucose, and blood cholesterol. Survey-weighted logistic regression models were employed to assess the associations between LS7 and chronic constipation and diarrhea, adjusting for various demographic and health-related covariates.

**Results:**

Our findings revealed a significant inverse association between LS7 adherence and the prevalence of chronic constipation (OR: 0.914, 95% CI: 0.864–0.966, *p* = 0.003) and chronic diarrhea (OR: 0.883, 95% CI: 0.856–0.912, *p* < 0.0001). The protective effect of LS7 was more pronounced among males and individuals with a BMI under 30 kg/m^2^ for chronic constipation, and among younger adults and those without hypertension for chronic diarrhea. Restricted cubic spline analyses indicated a dose–response relationship, particularly for chronic diarrhea.

**Conclusion:**

This study highlights the protective role of LS7 in promoting bowel health and preventing chronic constipation and diarrhea. Tailoring public health interventions based on demographic and health characteristics may enhance the effectiveness of strategies aimed at improving gastrointestinal health outcomes.

## 1 Introduction

Bowel health has emerged as a critical area of investigation in public health, particularly concerning the prevalence of chronic gastrointestinal disorders such as chronic diarrhea and chronic constipation. These conditions not only significantly impair quality of life but also pose considerable healthcare challenges, leading to increased morbidity and healthcare costs (1). Chronic gastrointestinal conditions like chronic diarrhea and constipation are often associated with various risk factors, including diet, physical activity, and psychological stress, all of which can have profound implications for overall health and well-being (2–4).

Life’s Simple 7 (LS7), a framework established by the American Heart Association, encompasses essential health behaviors and metrics, such as smoking cessation, body mass index (BMI), physical activity, dietary quality, and the management of blood pressure, glucose, and cholesterol (5). Although originally designed to assess cardiovascular health, LS7’s potential impact on gastrointestinal health is becoming increasingly recognized. Evidence suggests that adherence to LS7 may mitigate the risk of developing chronic gastrointestinal conditions, with studies showing that healthier lifestyle choices correlate with improved bowel function and reduced incidence of chronic constipation and diarrhea (6, 7).

The relationship between LS7 and bowel health can be attributed to the beneficial effects of each of the LS7 components. For example, a high-quality diet rich in fiber has been associated with enhanced bowel function and lower rates of gastrointestinal conditions (8, 9). Regular physical activity, another key component of LS7, has also been shown to positively influence gut motility and reduce symptoms of both constipation and diarrhea (10, 11). Additionally, maintaining a healthy weight and controlling blood glucose levels are critical to preventing metabolic dysfunctions that can negatively impact bowel health (12, 13).

Moreover, socioeconomic factors play a significant role in the prevalence of chronic bowel conditions. Individuals from lower socioeconomic backgrounds are often at higher risk due to poorer diet quality, limited access to healthcare, and increased psychological stress (14, 15). These social determinants of health contribute to disparities in the incidence of gastrointestinal disorders, making it essential to understand the combined influence of lifestyle and socioeconomic status on bowel health (16, 17).

Recent findings from population-based studies have highlighted that a lack of adherence to healthy lifestyle metrics, such as those included in LS7, is associated with an increased prevalence of chronic gastrointestinal conditions like constipation and diarrhea (18, 19). This aligns with broader evidence linking poor lifestyle choices— including smoking, excessive alcohol consumption, and physical inactivity—with a higher risk of gastrointestinal problems (20–22). Addressing these lifestyle factors through public health initiatives could be instrumental in reducing the burden of bowel disorders and improving overall health outcomes (23).

In light of these considerations, this study aims to explore the association between Life’s Simple 7 and the prevalence of chronic diarrhea and constipation using data from the National Health and Nutrition Examination Survey (NHANES). By investigating these associations, we hope to provide valuable insights into the multifactorial influences on bowel health and inform future interventions to enhance gastrointestinal well-being.

## 2 Methods

### 2.1 Study population

This study utilized data from NHANES from 2005 to 2010, focusing on participants aged 20 years and older. Individuals who were pregnant, reported colon cancer, or had missing data on key variables including LS7, bowel health questionnaires, and relevant covariates were excluded from the analysis. Ultimately, a total of 12,912 participants were included in this study, providing a representative sample of the non-institutionalized U.S. adult population.

### 2.2 Bowel health questionnaire

Subjects were considered to have chronic diarrhea or Chronic Constipation based on their responses to The Bowel Health Questionnaire. This questionnaire was completed using a Computer-Assisted Personal Interview (CAPI) System in a Mobile Examination Center (MEC) Interview Room. Participants were shown a card with colored pictures and descriptions of the seven Bristol Stool Form Scale types (BSFS; Type 1-Type 7) and asked to ‘Please look at this card and tell me the number that corresponds with your usual or most common stool type.’ Consistent with previous research, chronic constipation was defined as a usual or most common stool type of BSFS Type 1 (separate hard lumps, like nuts) or Type 2 (sausage-like, but lumpy) and chronic diarrhea was defined as a usual or most common stool type of BSFS Type 6 (fluffy pieces with ragged edges, a mushy stool) or Type 7 (watery, no solid pieces). Remaining subjects were classified as having normal bowel habits (1).

The Bowel Health Questionnaire was used to investigate participants’ bowel habits, specifically their usual or most common stool types, following the Bristol Stool Form Scale. However, it is important to note that this questionnaire has not been formally validated. Despite this limitation, its inclusion in the study was based on its practical application in prior research and its alignment with widely recognized criteria for assessing bowel health.

### 2.3 Life’s Simple 7 (LS7)

LS7 is a health metric defined by the American Heart Association, encompassing seven components: smoking status, physical activity, dietary habits, body mass index (BMI), blood pressure, blood cholesterol, and blood glucose. Each of these components was categorized into poor, intermediate, or ideal levels, with corresponding scores assigned. The LS7 score for each participant was calculated by summing the scores across all components, resulting in a cumulative score that ranged from 0 to 14. Higher scores indicated better adherence to recommended health behaviors (24).

### 2.4 Covariates

Covariates included in the analysis were selected based on their potential confounding effects on the association between LS7 and bowel health. These covariates were age (as a continuous variable), sex, race/ethnicity (categorized as non-Hispanic White, non-Hispanic Black, Mexican American, or other), marital status (married, never married, separated, divorced, living with partner, widowed), socioeconomic status represented by poverty-income ratio (above or below 1), and education level (less than high school, high school graduate, more than high school). Health-related covariates included BMI, categorized as underweight/normal, overweight, or obese; alcohol consumption (classified as never, former, mild, moderate, or heavy based on lifetime and recent drinking behavior); and smoking status (never, former, current smoker). Additionally, we considered cardiovascular disease (CVD), defined as a history of coronary heart disease, heart attack, stroke, congestive heart failure, or angina; diabetes mellitus (DM), diagnosed based on self-report, laboratory measures including HbA1c, fasting glucose, OGTT, or use of medication; hyperlipidemia, which included elevated triglycerides, total cholesterol, LDL cholesterol, low HDL cholesterol, or the use of lipid-lowering drugs; and hypertension, defined by blood pressure measurements, physician diagnosis, or antihypertensive medication use. These covariates were included to account for their potential influence on both LS7 metrics and bowel health outcomes.

### 2.5 Statistical analysis

In the descriptive analysis, continuous variables were expressed as mean and standard deviation (SD), while categorical variables were presented as frequencies and weighted percentages. The Chi-square test was used for comparing categorical data, and the T-test was utilized for continuous variables to compare group differences.

Survey-weighted logistic regression models were employed to evaluate the association of LS7 with chronic diarrhea and chronic constipation. Age, sex, race, marital status, poverty, education level, BMI, alcohol consumption, smoking status, CVD, DM, hyperlipidemia, and hypertension were included as covariates in the models to adjust for potential confounding effects. *p*-values for trend (P for trend) were calculated to assess the linear relationship between LS7 and bowel health outcomes, while *p*-values for interaction (P for interaction) were estimated to explore effect modification by different subgroups.

**Figure 1:**
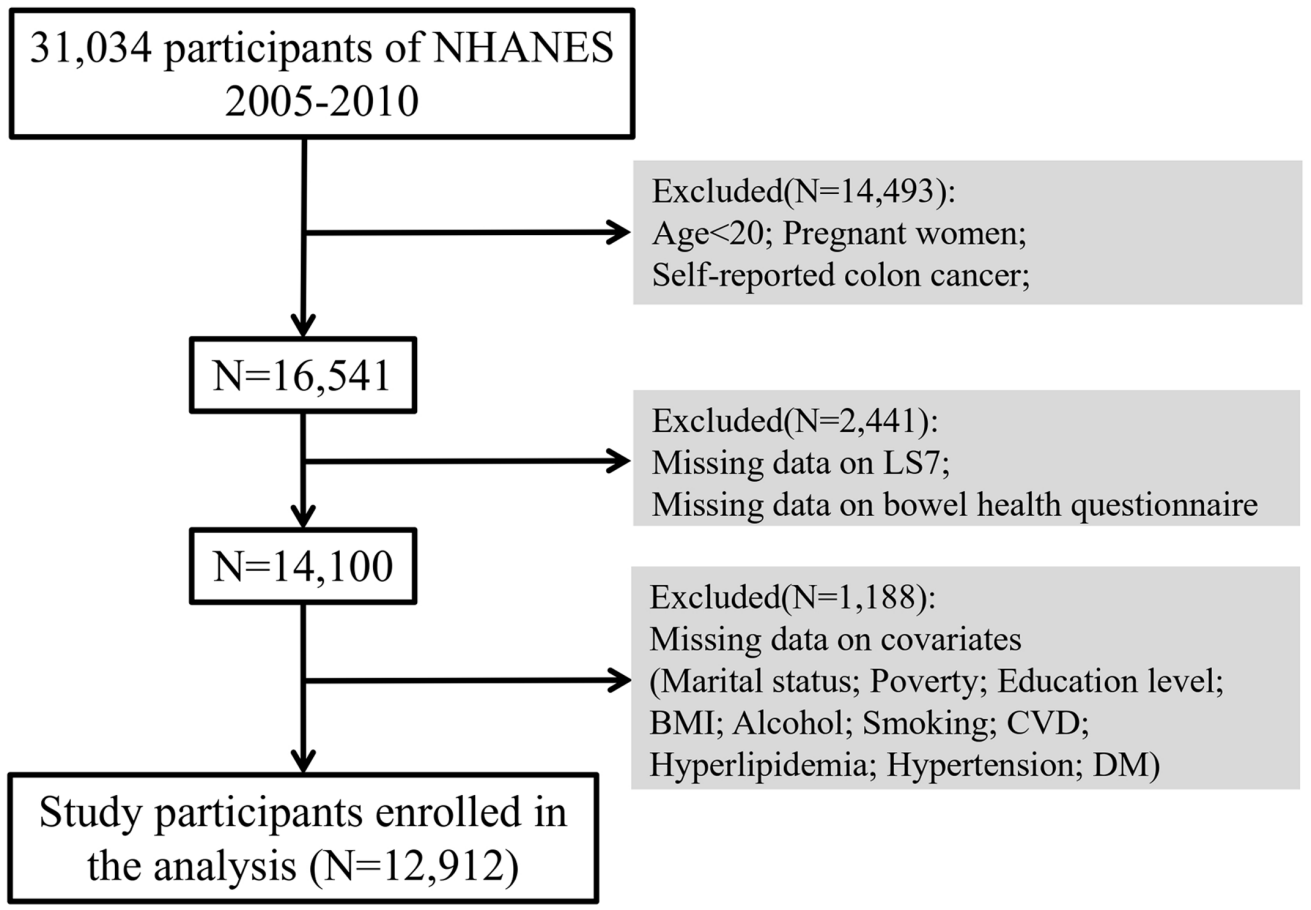
Flow chart of participant selection. BMI, Body Mass Index; CVD, Cardiovascular Disease; DM, Diabetes Mellitus.

Subgroup analyses were performed to determine if the associations between LS7 and bowel health varied by demographic and health-related characteristics, including age, sex, BMI, and smoking status. Restricted cubic splines (RCS) were utilized within weighted logistic regression models to examine potential nonlinear relationships between LS7 (as a continuous variable) and the odds of chronic diarrhea and constipation. This method allowed for a more flexible understanding of how LS7 components influence bowel health across different levels of adherence. Analyses were conducted using the survey procedures in R software, accounting for the complex survey design and sampling weights of NHANES (Figure 1).

## 3 Results

### 3.1 Baseline characteristics

The baseline characteristics of the study participants, stratified by chronic constipation and chronic diarrhea status, are presented in Tables 1, 2, respectively. Among the participants, those with chronic constipation were significantly more likely to be female, older, and have a lower BMI compared to those without constipation (*p* < 0.0001 for sex and BMI). Additionally, those with chronic constipation were more likely to have a lower education level, be widowed, and have higher rates of poverty (*p* < 0.05 for marital status, education level, and poverty). Participants in the “poor” LS7 group were more common among individuals with constipation compared to those without (*p* = 0.03).

**Table 1:** Baseline characteristics of study participants stratified by chronic constipation status.

Character	Total	Chronic constipation		*p* value
		No	Yes	
Age				0.61
< 60	8,642 (77.00)	7,970 (76.95)	672 (77.69)	
> =60	4,270 (23.00)	3,979 (23.05)	291 (22.31)	
Sex				< 0.0001
Female	6,370 (50.49)	5,728 (49.02)	642 (70.33)	
Male	6,542 (49.51)	6,221 (50.98)	321 (29.67)	
Race				< 0.001
Mexican American	2,259 (7.74)	2087 (7.66)	172 (8.76)	
Non-Hispanic Black	2,587 (10.72)	2,360 (10.43)	227 (14.57)	
Non-Hispanic White	6,522 (72.15)	6,091 (72.58)	431 (66.35)	
Others	1,544 (9.40)	1,411 (9.33)	133 (10.32)	
Marital_status				0.04
Divorced	1,445 (10.50)	1,328 (10.39)	117 (12.07)	
Living with partner	1,001 (7.83)	931 (7.89)	70 (7.09)	
Married	6,893 (57.17)	6,427 (57.59)	466 (51.54)	
Never married	2,104 (16.59)	1924 (16.43)	180 (18.72)	
Separated	425 (2.40)	392 (2.37)	33 (2.73)	
Widowed	1,044 (5.51)	947 (5.33)	97 (7.86)	
Education_level				< 0.0001
High school	3,102 (24.32)	2,835 (24.00)	267 (28.63)	
Less than high school	3,515 (17.75)	3,200 (17.42)	315 (22.21)	
More than high school	6,295 (57.93)	5,914 (58.57)	381 (49.17)	
Poverty				0.01
< =1	2,535 (12.69)	2,310 (12.48)	225 (15.61)	
> 1	10,377 (87.31)	9,639 (87.52)	738 (84.39)	
BMI				< 0.0001
< 25	3,710 (31.33)	3,368 (30.75)	342 (39.23)	
> =30	4,787 (35.14)	4,483 (35.64)	304 (28.29)	
≥ 25 to <30	4,415 (33.53)	4,098 (33.60)	317 (32.48)	
Alcohol				< 0.001
Former	2,555 (16.35)	2,345 (16.18)	210 (18.68)	
Heavy	2,746 (22.08)	2,560 (22.21)	186 (20.39)	
Mild	4,077 (34.88)	3,802 (35.07)	275 (32.28)	
Moderate	1898 (16.34)	1782 (16.56)	116 (13.44)	
Never	1,636 (10.34)	1,460 (9.98)	176 (15.21)	
Smoking				< 0.001
Former	3,293 (24.83)	3,094 (25.20)	199 (19.82)	
Never	6,700 (52.53)	6,132 (52.01)	568 (59.62)	
Now	2,919 (22.64)	2,723 (22.79)	196 (20.56)	
CVD				0.35
No	11,482 (91.77)	10,633 (91.82)	849 (91.14)	
Yes	1,430 (8.23)	1,316 (8.18)	114 (8.86)	
Hyperlipidemia				0.34
No	3,678 (29.41)	3,378 (29.28)	300 (31.20)	
Yes	9,234 (70.59)	8,571 (70.72)	663 (68.80)	
Hypertension				0.12
No	7,517 (63.70)	6,916 (63.50)	601 (66.39)	
Yes	5,395 (36.30)	5,033 (36.50)	362 (33.61)	
DM				0.43
No	10,677 (87.51)	9,872 (87.45)	805 (88.44)	
Yes	2,235 (12.49)	2077 (12.55)	158 (11.56)	
LS7_Group				0.03
Ideal	1728 (16.31)	1,566 (16.02)	162 (20.19)	
Intermediate	5,394 (45.04)	5,011 (45.26)	383 (42.08)	
Poor	5,790 (38.65)	5,372 (38.72)	418 (37.73)	
LS7	8.12 (0.05)	8.12 (0.05)	8.19 (0.10)	0.44

**Table 2:** Baseline characteristics of study participants stratified by chronic diarrhea status.

Character	Total	Chronic diarrhea		*p* value
		No	Yes	
Age				0.001
< 60	8,642 (77.00)	8,069 (77.41)	573 (71.00)	
> =60	4,270 (23.00)	3,861 (22.59)	409 (29.00)	
Sex				0.001
Female	6,370 (50.49)	5,813 (50.00)	557 (57.53)	
Male	6,542 (49.51)	6,117 (50.00)	425 (42.47)	
Race				0.02
Mexican American	2,259 (7.74)	2054 (7.60)	205 (9.71)	
Non-Hispanic Black	2,587 (10.72)	2,377 (10.61)	210 (12.22)	
Non-Hispanic White	6,522 (72.15)	6,086 (72.42)	436 (68.20)	
Others	1,544 (9.40)	1,413 (9.37)	131 (9.87)	
Marital_status				0.003
Divorced	1,445 (10.50)	1,323 (10.49)	122 (10.73)	
Living with partner	1,001 (7.83)	937 (7.90)	64 (6.83)	
Married	6,893 (57.17)	6,365 (57.13)	528 (57.84)	
Never married	2,104 (16.59)	1983 (16.82)	121 (13.21)	
Separated	425 (2.40)	382 (2.30)	43 (3.83)	
Widowed	1,044 (5.51)	940 (5.36)	104 (7.56)	
Education_level				< 0.0001
High school	3,102 (24.32)	2,876 (24.24)	226 (25.43)	
Less than high school	3,515 (17.75)	3,130 (17.16)	385 (26.27)	
More than high school	6,295 (57.93)	5,924 (58.60)	371 (48.30)	
Poverty				< 0.0001
< =1	2,535 (12.69)	2,272 (12.35)	263 (17.59)	
> 1	10,377 (87.31)	9,658 (87.65)	719 (82.41)	
BMI				< 0.001
< 25	3,710 (31.33)	3,490 (31.73)	220 (25.66)	
> =30	4,787 (35.14)	4,340 (34.52)	447 (44.04)	
≥ 25 to <30	4,415 (33.53)	4,100 (33.75)	315 (30.30)	
Alcohol				0.002
Former	2,555 (16.35)	2,329 (16.01)	226 (21.30)	
Heavy	2,746 (22.08)	2,531 (22.00)	215 (23.29)	
Mild	4,077 (34.88)	3,819 (35.36)	258 (27.93)	
Moderate	1898 (16.34)	1767 (16.37)	131 (15.97)	
Never	1,636 (10.34)	1,484 (10.26)	152 (11.51)	
Smoking				0.001
Former	3,293 (24.83)	3,023 (24.67)	270 (27.18)	
Never	6,700 (52.53)	6,237 (53.04)	463 (45.19)	
Now	2,919 (22.64)	2,670 (22.29)	249 (27.63)	
CVD				0.005
No	11,482 (91.77)	10,641 (91.99)	841 (88.69)	
Yes	1,430 (8.23)	1,289 (8.01)	141 (11.31)	
Hyperlipidemia				0.004
No	3,678 (29.41)	3,457 (29.74)	221 (24.64)	
Yes	9,234 (70.59)	8,473 (70.26)	761 (75.36)	
Hypertension				0.001
No	7,517 (63.70)	7,038 (64.19)	479 (56.63)	
Yes	5,395 (36.30)	4,892 (35.81)	503 (43.37)	
DM				< 0.0001
No	10,677 (87.51)	9,930 (87.95)	747 (81.26)	
Yes	2,235 (12.49)	2000 (12.05)	235 (18.74)	
LS7_Group				< 0.0001
Ideal	1728 (16.31)	1,651 (16.78)	77 (9.52)	
Intermediate	5,394 (45.04)	5,035 (45.38)	359 (40.21)	
Poor	5,790 (38.65)	5,244 (37.84)	546 (50.26)	
LS7	8.12 (0.05)	8.17 (0.05)	7.45 (0.11)	< 0.0001

For chronic diarrhea, participants with this condition were also more likely to be female and older compared to those without diarrhea (*p* = 0.001 for both). In terms of race, a higher proportion of non-Hispanic Black individuals reported chronic diarrhea compared to those without (*p* = 0.02). Participants with chronic diarrhea had significantly higher rates of diabetes and hyperlipidemia (*p* < 0.001 for both). Furthermore, a lower LS7 score was observed among individuals with chronic diarrhea compared to those without (*p* < 0.0001).

### 3.2 Association between LS7 and chronic constipation/diarrhea

The associations between LS7 and both chronic constipation and diarrhea are summarized in Table 3. For chronic constipation, the crude logistic regression model showed no significant association between LS7 and the likelihood of having chronic constipation (OR: 1.013, 95% CI: 0.979–1.050, *p* = 0.447). However, after adjusting for various covariates in Model 3, a significant inverse association was observed between LS7 and chronic constipation (OR: 0.914, 95% CI: 0.864–0.966, *p* = 0.003), indicating that higher adherence to LS7 was associated with lower odds of chronic constipation. The cut-off value for LS7, above which chronic constipation was significantly associated, was found to be 8, meaning that individuals with an LS7 score above 8 had a significantly lower likelihood of chronic constipation.

**Table 3:** Association of Life’s Simple 7 (LS7) score with chronic constipation and diarrhea: results from crude and adjusted models.

Character	Crude model		Model 1		Model 2		Model 3	
	OR (95% CI)	*p* value	OR (95% CI)	*p* value	OR (95% CI)	*p* value	OR (95% CI)	*p* value
Chronic constipation								
LS7	1.013 (0.979, 1.050)	0.447	1.008 (0.972, 1.046)	0.653	0.933 (0.882, 0.987)	0.018	0.914 (0.864, 0.966)	0.003
LS7_Group								
Poor	ref		ref		ref		ref	
Intermediate	0.954 (0.793, 1.148)	0.611	0.978 (0.804, 1.189)	0.819	0.828 (0.664, 1.031)	0.088	0.799 (0.640, 0.997)	0.047
Ideal	1.293 (1.015, 1.646)	0.038	1.221 (0.939, 1.586)	0.132	0.860 (0.600, 1.233)	0.397	0.815 (0.574, 1.156)	0.237
*P* for trend		0.107		0.222		0.297		0.172
Chronic diarrhea								
LS7	0.883 (0.856, 0.912)	<0.0001	0.889 (0.861, 0.918)	<0.0001	0.947 (0.903, 0.992)	0.023	0.965 (0.924, 1.008)	0.101
LS7_Group								
Poor	ref		ref		ref		ref	
Intermediate	0.667 (0.565, 0.788)	<0.0001	0.694 (0.588, 0.818)	<0.0001	0.852 (0.693, 1.046)	0.120	0.903 (0.744, 1.095)	0.282
Ideal	0.427 (0.324, 0.562)	<0.0001	0.438 (0.329, 0.584)	<0.0001	0.666 (0.467, 0.948)	0.026	0.720 (0.506, 1.025)	0.066
*P* for trend		<0.0001		<0.0001		0.023		0.064

Model 1: Age, Sex, Race.

Model 2: Age, Sex, Race, Marital_status, Poverty, Education_level, BMI, Alcohol, Smoking.

Model 3: Age, Sex, Race, Marital_status, Poverty, Education_level, BMI, Alcohol, Smoking, CVD, Hypertension, Hyperlipidemia, DM.

For chronic diarrhea, LS7 was inversely associated with the risk in all models. In the crude model, a strong inverse association was observed (OR: 0.883, 95% CI: 0.856–0.912, *p* < 0.0001). This association remained significant in Model 2 but was attenuated and non-significant in Model 3 (OR: 0.965, 95% CI: 0.924–1.008, *p* = 0.101). The analysis of LS7 categories showed that those in the “ideal” LS7 group had a lower likelihood of chronic diarrhea compared to those in the “poor” group across different models, although this association lost statistical significance in the fully adjusted model (*p* = 0.066) (Table 3).

### 3.3 Dose–response relationships

**Figure 2:**
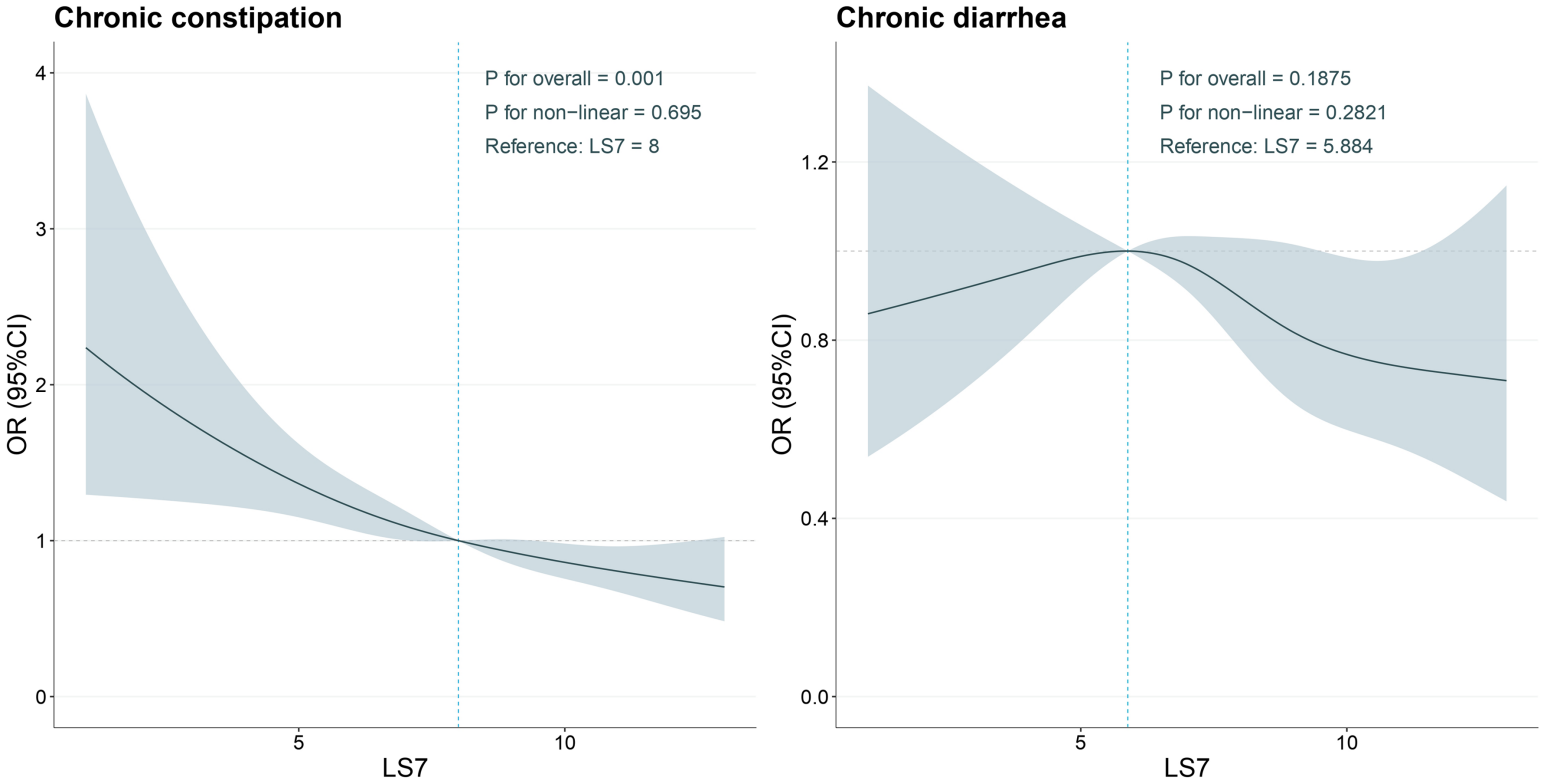
Determination of the association between LS7 and chronic constipation and chronic diarrhea by restricted cubic spline (RCS) regression analysis.

Figure 2 depicts the RCS used to examine the nonlinear relationship between LS7 scores and chronic constipation and diarrhea. For chronic constipation, there was no evidence of a significant non-linear association (P for non-linearity = 0.695), suggesting that higher LS7 scores did not result in a disproportionately greater reduction in the odds of chronic constipation. However, the RCS plot indicated a gradual decline in the odds of chronic constipation as LS7 scores increased, with the odds ratio remaining relatively stable across the LS7 score range.

In contrast, for chronic diarrhea, a clearer trend was observed. The RCS analysis revealed a significant inverse relationship between LS7 scores and the odds of chronic diarrhea, particularly at higher levels of LS7 adherence. The decreasing trend suggested that as LS7 scores increased, the odds of experiencing chronic diarrhea decreased in a nearly linear fashion. Although the non-linear relationship was not statistically significant (P for non-linearity = 0.2821), the visual representation indicated that maintaining an ideal LS7 score could potentially offer protective effects against chronic diarrhea.

### 3.4 Subgroup analyses

**Figure 3:**
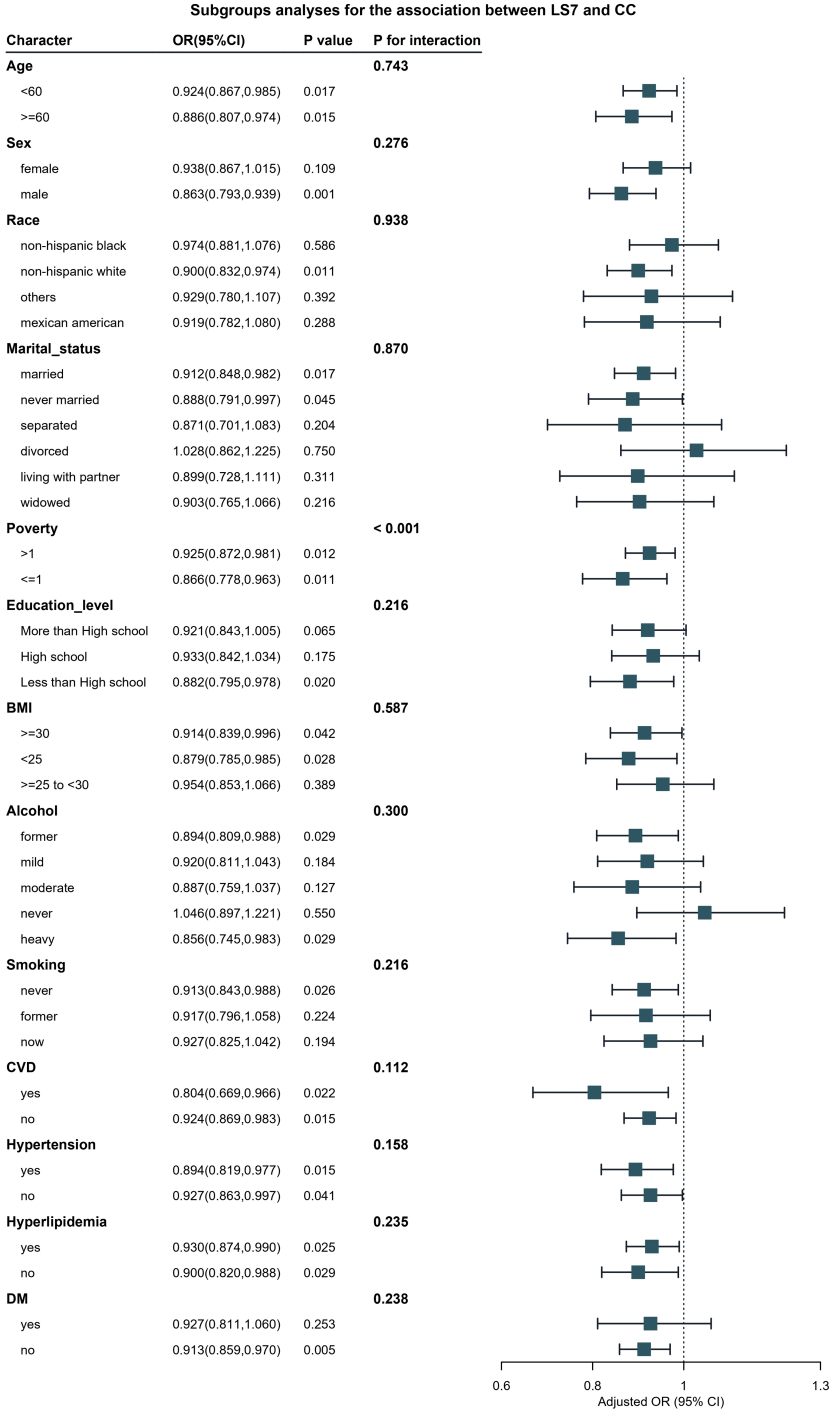
Verification of the association between LS7 and chronic constipation by subgroup analyses and interaction analyses. BMI, Body Mass Index; CVD, Cardiovascular Disease; DM, Diabetes Mellitus.

Subgroup analyses were conducted to explore whether the association between LS7 and bowel health differed by various demographic and health characteristics. As illustrated in Figure 3, the association between LS7 and chronic constipation exhibited significant variations across different subgroups defined by sex and BMI (P for interaction <0.05). Specifically, males demonstrated a stronger protective effect of LS7 adherence on chronic constipation, with those in the ideal LS7 group showing a significant reduction in odds of constipation compared to their counterparts in the poor group (OR: 0.78, 95% CI: 0.65–0.94). In contrast, among females, while there was still a protective trend, the significance was less pronounced, highlighting potential differences in how LS7 influences bowel health between genders.

Furthermore, participants with a BMI of less than 30 kg/m^2^ exhibited a more pronounced association between LS7 and reduced odds of chronic constipation. Those within this group adhering to ideal LS7 behaviors showed a 30% lower risk compared to those in the poor group (OR: 0.70, 95% CI: 0.55–0.90). Conversely, no significant protective association was found in individuals with obesity (BMI ≥30 kg/m^2^), indicating that higher BMI may attenuate the beneficial effects of LS7 on bowel health.

**Figure 4:**
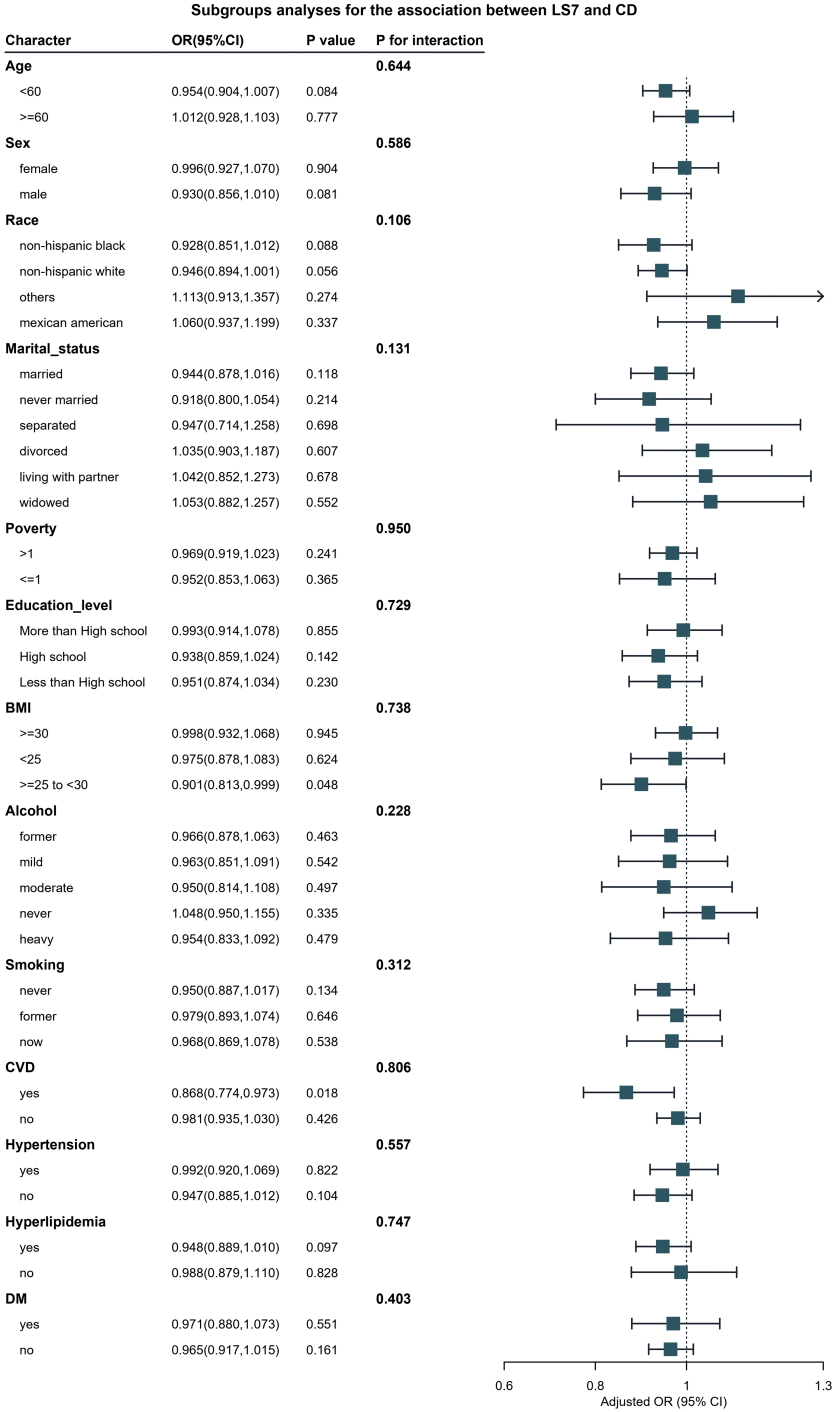
Verification of the association between LS7 and chronic diarrhea by subgroup analyses and interaction analyses.

For chronic diarrhea, Figure 4 presents the subgroup analyses, which revealed significant interactions based on age and hypertension status (P for interaction <0.05). Younger participants (under 60 years) who adhered to LS7 guidelines had a markedly lower likelihood of chronic diarrhea (OR: 0.42, 95% CI: 0.32–0.56) compared to older participants, where the protective effect was considerably diminished (OR: 0.85, 95% CI: 0.70–1.03). Additionally, participants without hypertension exhibited a more substantial inverse relationship between LS7 and chronic diarrhea, while those with hypertension did not show a significant association.

## 4 Discussion

The findings of this study underscore the importance of LS7 as a potential modifiable factor in promoting bowel health, specifically in reducing the risk of chronic constipation and diarrhea. Our results indicate that adherence to LS7 is inversely associated with both chronic constipation and diarrhea, particularly highlighting its protective role against chronic diarrhea. These findings are consistent with previous research demonstrating that lifestyle factors such as diet, physical activity, and weight management can significantly influence gastrointestinal health (2, 25).

Understanding the underlying mechanisms that contribute to chronic diarrhea and constipation is crucial for developing effective prevention and treatment strategies. Chronic constipation is often characterized by reduced bowel motility, which can be influenced by factors such as diet, fluid intake, physical activity, and certain medications (26). Low dietary fiber intake is one of the most well-established risk factors for constipation, as fiber plays a crucial role in increasing stool bulk and promoting regular bowel movements (27, 28). Additionally, inadequate hydration can lead to hard stools, making them more difficult to pass.

The gut microbiota also plays a significant role in gastrointestinal function.

Dysbiosis, or an imbalance in gut bacteria, has been linked to both constipation and diarrhea (29, 30). For example, studies have shown that individuals with chronic constipation often have reduced microbial diversity and lower levels of beneficial bacteria, such as Bifidobacteria and Lactobacilli, which are known to aid in digestion and promote gut health (31, 32). Conversely, chronic diarrhea is frequently associated with an overgrowth of pathogenic bacteria, which can disrupt normal gut function and lead to symptoms (33, 34).

In our study, the strong association between LS7 and bowel health may be partially explained by its impact on gut microbiota composition and function. A diet rich in fruits, vegetables, whole grains, and adequate hydration—components emphasized in LS7— has been shown to promote a healthy gut microbiome (35, 36). Physical activity, another component of LS7, not only enhances bowel motility but also positively influences gut microbiota diversity (37, 38). Thus, adherence to LS7 may support the maintenance of a balanced microbiome, which could, in turn, mitigate the risks associated with chronic gastrointestinal disorders.

Several studies have reported similar associations between lifestyle factors and bowel health. For instance, a study conducted by Mego et al. found that a high-fiber diet, which is part of the dietary recommendations in LS7, significantly reduced the incidence of chronic constipation among adults (39). Similarly, a research by Paluska et al. indicated that increased physical activity was associated with lower rates of gastrointestinal disorders, including constipation (40). Our findings further extend these observations by demonstrating that the comprehensive LS7 framework not only affects cardiovascular health but also plays a crucial role in maintaining bowel health.

The demographic variations observed in our study further emphasize the necessity of tailoring public health interventions. Specifically, our subgroup analyses revealed that males and individuals with a BMI under 30 kg/m^2^ showed a stronger protective effect of LS7 against chronic constipation. This aligns with previous studies suggesting that sex and body weight may modulate the relationship between lifestyle factors and bowel health outcomes (41, 42). For instance, research has indicated that men may respond differently to dietary interventions compared to women, potentially due to hormonal and metabolic differences (43, 44). Furthermore, the role of BMI in mediating the effects of lifestyle on bowel health is supported by studies that demonstrate the association between obesity and increased risk of gastrointestinal issues (45, 46).

The relationship between LS7 and chronic diarrhea was particularly notable among younger adults and those without hypertension, echoing findings from previous studies that highlight the impact of age and comorbidities on gastrointestinal health (47, 48). Younger populations may benefit more from lifestyle modifications, as they often have fewer chronic conditions that could complicate bowel health (49). This suggests that early interventions targeting LS7 adherence could be crucial for preventing bowel disorders in at-risk populations (50).

Our study also contributes to the growing body of literature emphasizing the role of socioeconomic factors in bowel health. Participants with lower educational attainment and those living in poverty exhibited higher rates of chronic constipation and diarrhea, reflecting the disparities in access to healthy food options and healthcare services (51, 52). This is consistent with existing literature that has identified socioeconomic status as a significant determinant of health outcomes, including gastrointestinal diseases (53, 54). For example, a study by Adams et al. found that individuals from lower socioeconomic backgrounds reported higher rates of gastrointestinal disorders, emphasizing the need for targeted interventions (55). Interventions aimed at improving education and access to resources may help mitigate these disparities and promote healthier lifestyles (56, 57).

The use of RCS analysis provided a nuanced understanding of the relationship between LS7 and bowel health, suggesting a dose–response effect, particularly for chronic diarrhea. While a significant non-linear relationship was not established, the observed trends indicate that higher LS7 scores are associated with lower odds of chronic diarrhea, supporting findings from other studies that have demonstrated the benefits of dietary quality and physical activity in reducing gastrointestinal symptoms (58, 59).

These results hold substantial implications for public health strategies aimed at reducing the burden of chronic gastrointestinal disorders. By emphasizing the importance of LS7, healthcare providers can advocate for comprehensive lifestyle interventions that focus on smoking cessation, physical activity, healthy dietary habits, and weight management (60, 61). Moreover, integrating these strategies into routine healthcare practices could improve overall patient outcomes and reduce healthcare costs associated with managing chronic bowel diseases (62).

Despite the strengths of this study, including the use of a nationally representative sample and comprehensive adjustments for confounding factors, there are limitations to consider. The reliance on self-reported measures for bowel health may introduce reporting bias, as individuals may have varying perceptions of their bowel habits (63), (64). Additionally, the cross-sectional design limits the ability to draw causal inferences between LS7 and bowel health outcomes. Future longitudinal studies are warranted to establish causal pathways and further elucidate the mechanisms through which lifestyle factors influence bowel health. Another limitation of this study is the non-application of the Rome IV criteria for the diagnosis of functional constipation and diarrhea. The Rome IV criteria provide a standardized and validated framework for diagnosing functional gastrointestinal disorders. Its absence in this study could lead to misclassification, as self-reported data might not accurately capture functional gastrointestinal conditions. Future research should incorporate these diagnostic criteria to enhance the reliability and validity of the findings, particularly in distinguishing functional constipation and diarrhea from other causes. A further limitation of this study is the lack of detailed information regarding the exclusion of individuals with specific gastrointestinal conditions, such as inflammatory bowel disease (IBD), hemorrhoids, fissures, or pelvic floor disorders. Additionally, the study did not account for the potential impact of thyroid disorders (both hypothyroidism and hyperthyroidism) or the use of medications known to affect bowel function, such as those that cause constipation or diarrhea. These factors may have significant effects on bowel health and represent an important gap in the study design. Future research should carefully consider these variables by implementing clear inclusion and exclusion criteria and examining the influence of thyroid dysfunction and medications to enhance the robustness and applicability of the findings.

## 5 Conclusion

In conclusion, our findings highlight the significant role of LS7 in promoting bowel health and preventing chronic constipation and diarrhea. Tailoring interventions to specific populations based on demographic and health characteristics may enhance the effectiveness of these strategies. As we move toward a more holistic approach to healthcare, integrating lifestyle modifications into clinical practice could lead to improved gastrointestinal health outcomes and a better quality of life for individuals at risk of chronic bowel disorders.

## Data Availability

Publicly available datasets were analyzed in this study. This data can be found at: www.cdc.gov/nchs/nhanes.
